# The Complex Role of the Microbiome in Non-Small Cell Lung Cancer Development and Progression

**DOI:** 10.3390/cells12242801

**Published:** 2023-12-08

**Authors:** Vanessa G. P. Souza, Aisling Forder, Michelle E. Pewarchuk, Nikita Telkar, Rachel Paes de Araujo, Greg L. Stewart, Juliana Vieira, Patricia P. Reis, Wan L. Lam

**Affiliations:** 1 British Columbia Cancer Research Institute, Vancouver, BC V5Z 1L3, Canada; 2 Molecular Oncology Laboratory, Experimental Research Unit, School of Medicine, São Paulo State University (UNESP), Botucatu 18618-687, SP, Brazilpatricia.reis@unesp.br (P.P.R.); 3 British Columbia Children’s Hospital Research Institute, Vancouver, BC V5Z 4H4, Canada; 4 Department of Surgery and Orthopedics, Faculty of Medicine, São Paulo State University (UNESP), Botucatu 18618-687, SP, Brazil

**Keywords:** lung cancer, microbiome, immunotherapy, tumor microenvironment (TME)

## Abstract

In recent years, there has been a growing interest in the relationship between microorganisms in the surrounding environment and cancer cells. While the tumor microenvironment predominantly comprises cancer cells, stromal cells, and immune cells, emerging research highlights the significant contributions of microbial cells to tumor development and progression. Although the impact of the gut microbiome on treatment response in lung cancer is well established, recent investigations indicate complex roles of lung microbiota in lung cancer. This article focuses on recent findings on the human lung microbiome and its impacts in cancer development and progression. We delve into the characteristics of the lung microbiome and its influence on lung cancer development. Additionally, we explore the characteristics of the intratumoral microbiome, the metabolic interactions between lung tumor cells, and how microorganism-produced metabolites can contribute to cancer progression. Furthermore, we provide a comprehensive review of the current literature on the lung microbiome and its implications for the metastatic potential of tumor cells. Additionally, this review discusses the potential for therapeutic modulation of the microbiome to establish lung cancer prevention strategies and optimize lung cancer treatment.

## 1. Introduction

Lung cancer is widely recognized as a leading cause of cancer-related mortality worldwide, with an estimated 2.2 million new cancer cases and 1.8 million deaths in 2020 [1]. Although there have been notable advancements in targeted therapies and immunotherapies for lung cancer in recent years, the disease’s overall survival rates are still low (<20%) [2,3]. It is forecasted that lung cancer will be the most expensive cancer regarding diagnoses and treatment in the next 30 years, with a projected cost of USD 3.9 trillion [4]. As a result, it is critical to comprehend the root causes and risk factors associated with it as part of public health initiatives. While it is well-established that most lung cancer cases are attributed to smoking, other elements, including exposure to radon gas, asbestos, air pollution, and chronic infections, have been implicated in its development, further emphasizing the multifaceted and complex nature of this disease [5,6]. To address this ongoing critical public health crisis, comprehensive insights into all the underlying causes of lung cancer are necessary.

**Figure 1:**
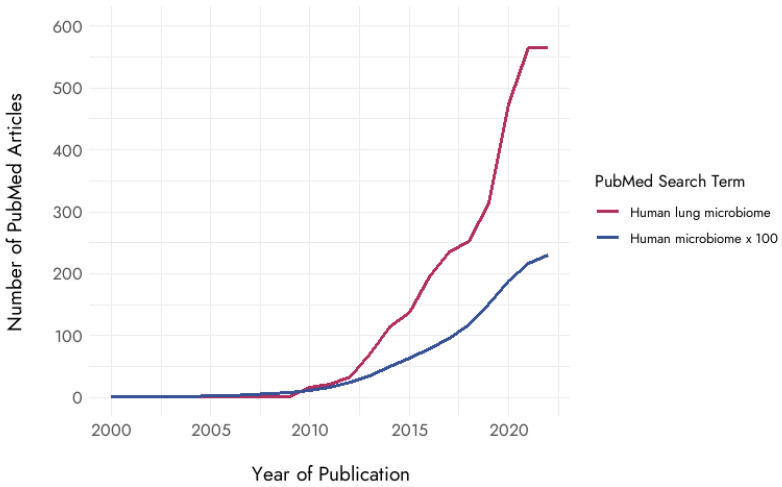
Trends in publications on the human lung microbiome (2000–2021). This figure illustrates the number of papers published on the human lung microbiome from 2000 to 2021. Data source: PubMed (accessed on 28 September 2023). Pubmed search terms: blue = human microbiome; pink = human lung microbiome.

In recent years, with the rapid advancement of sequencing technology, there has been a surge of interest in understanding the lung microbiome (Figure 1). The microbiome is defined as the full complement of microbes such as bacteria, fungi, viruses, protozoa, and their related genes and genomes, as well as metabolites, while the microbiota refers to the assemblage of microorganisms present in a defined environment [7]. Traditionally, healthy lungs were believed to be sterile—except for infections—since conventional culture techniques rarely isolated bacteria from them. However, with the advent of next-generation sequencing (NGS) technologies, a wide variety of bacterial DNA has been commonly detected in the lower respiratory tract of healthy individuals [8,9,10]. The lung microbiome is relatively low in biomass, with 10^3^ to 10^5^ bacteria per gram of tissue in healthy lungs [11]. The upper and lower respiratory tracts differ in their microbial composition and biomass [12]. Although the lung microbiota is relatively dynamic due to the immigration and elimination of microbiomes through aspiration, coughing, or mucociliary clearance [12], studies analyzing the microbial composition of healthy lungs indicate that the core lung microbiota include mainly *Actinobacteria*, *Bacteroidetes*, *Firmicutes*, and *Proteobacteria* families [13,14,15]. Furthermore, *Prevotella*, *Veillonella*, and *Streptococcus*, which are usually found in oral microbiota, have been identified in the lungs of most healthy individuals [8,9,12,13,14,16].

The lung microbiota play an important role in maintaining lung homeostasis and immune tolerance that protects the host from undesired inflammatory response [17]. Recognizing its pivotal contribution to maintaining lung homeostasis, the composition of the lung microbiota emerges as a valuable indicator for monitoring lung health status [18,19]. Recently, there has been evidence of a lung microbiome, and the microbiome’s alterations were found to be linked with disease states, such as exacerbations in chronic obstructive pulmonary disease (COPD) [20]. Dysbiosis, which is defined as deviation from a normal microbial composition, is associated with various adverse biological occurrences, occasionally yielding clinical implications. Within the lung context, dysbiosis holds substantial sway over the onset and advancement of respiratory diseases [12], such as asthma [21,22], cystic fibrosis [23,24], and acute respiratory distress syndrome [25].

Recent advances in the field of the gut microbiome have revealed how the gut microbiota can modulate antitumor immunity and impact the efficacy of cancer immunotherapies, particularly immune checkpoint inhibitors (ICIs) [26,27]. In patients with non-small cell lung cancer (NSCLC), a strong correlation has been demonstrated between gut microbiome diversity and responses to anti-PD-1 immunotherapy [28]. Furthermore, it has been shown that antibiotics are associated with decreased survival and attenuated responses to ICI in patients with advanced NSCLC [29].

While the involvement of the gut microbiome in treatment response has been well documented in the development and immunotherapy of lung cancer [30,31], recent studies suggest that the microbiota in the lungs also play a role in lung cancer development [32,33,34,35]. Microorganisms such as bacteria, viruses, and fungi have emerged as pivotal players in the complex interplay of factors contributing to cancer initiation, promotion, and progression [36] and lung cancer development [32,33]. Studies have reported that the levels of *Actinomyces*, *Veillonella*, *Streptococcus*, *Megasphaera*, and *Mycobacterium* were more abundant in patients with lung cancer compared with healthy individuals [37,38,39]. *Prevotella* and *Veillonella* were most strongly associated with NSCLC, and *Veillonella* significantly promoted the progression of lung cancer [37]. Oral bacteria such as *Streptococcus* spp. and *Veillonella* spp. were enriched in the lower respiratory tract of patients with lung cancer, which was associated with upregulation of the ERK and PI3K signaling [16]. The analysis of the community compositions of patients with lung cancer with or without emphysema showed a significantly lower abundance of Proteobacteria and a higher prevalence of Firmicutes (*Streptococcus*) and Bacteroidetes (*Prevotella*), compared to patients with emphysema only [40]. The genera *Veillonella* and *Megasphaera* exhibited relatively elevated levels in patients with lung cancer, suggesting their potential as biomarkers in lung cancer [39].

The promising field of microbial interactions within the tumor microenvironment (TME) presents exciting opportunities to comprehend the novel mechanisms of lung cancer progression. Through a comprehensive understanding of the intricate relationships between microbial and lung tumor cells, we can gain valuable insights into the potential crosstalk that influences tumor growth, immune responses, and therapeutic options.

Although previous studies have investigated the relationship between lung microbiota and lung cancer [32,33,41], our article offers novel insights by specifically highlighting the characteristics of the lung microbiome, the relationship between the lung microbiome and lung cancer risk, and the modes of interaction between lung microbiota and the host immune system. Additionally, we explore the metabolic interactions between lung tumor cells and microbial cells within the TME, as well as the functional effects thereof on lung cancer progression. We also discuss the possibilities of therapeutic modulation of the microbiome, aiming at the establishment of lung cancer prevention strategies and the optimization of lung cancer treatment.

## 2. Lung Microbiome

Historically, it was thought that healthy lungs were sterile, but culture-independent sequencing methods have revealed a variety of microbial communities in the lower respiratory tract [42]. These communities have been collectively termed as the lung microbiome, and refer to the collection of microorganisms (including bacteria, archaea, lower and higher eukaryotes, and viruses), and their genetic material that resides in an individual’s lung at a given moment in time [43,44]. It is known that the microbiome plays an important role in human health and disease by modulating the host’s innate and adaptive immune system, immune responses, and metabolism, and by protecting from invading pathogens [45,46]. A healthy lung microbiome shows a rich, dynamic, transient, and diverse bacterial community that is present in a low abundance, being characterized mainly by phyla *Firmicutes* and *Bacteroidetes*, and genera such as *Prevotella*, *Veillonella*, and *Streptococcus* [46,47]. Its composition is determined by the balance of three factors: (1) Microbial immigration into the airways driven by inhalation of microbes from air leading to microaspiration of the upper respiratory tract (URT) and oral cavity followed by direct dispersion along the airway mucosal surface. (2) The elimination of microbes from the airways by mucociliary clearance, coughing, and host immune defense systems (both innate and adaptive). (3) The relative reproduction rates of its community members found in the airways, which is determined by the regional growth conditions, including pH, temperature, oxygen tension, and nutrient availability, as well as local microbial competition, host epithelial cell interactions, and activation of host inflammatory cells [46]. It has been suggested that the URT contributes to the major source of lung microbiota since there is a close resemblance between upper and lower respiratory tract (LRT) microbiome composition in healthy individuals [21].

Moreover, bacterial levels are higher in the more proximal pulmonary regions, and there are also modest regional differences, suggesting differential clearance and potentially limited local replication [47]. In healthy lungs, the balance between the dispersal of microbes from the URT and clearance of lung microbial community members via local defense mechanisms is considered the major determinant of lung characteristics, whereas local bacterial reproduction most probably plays a minor role [21]. It may also be that lung microbiome composition in healthy individuals could be best attributed to the neutral dispersal of microbes from the oropharynx rather than active local bacterial selection in the lungs [48].

Many factors can influence the lung microbiome and cause microbiota dysbiosis. The use of some medications, such as antibiotics, steroids, and metformin, has a role in contributing to dysbiosis, which may have an impact on the disease states in the lungs [12,49]. Dysbiosis in the URT through aspirations, and inhalation of aerosols of microbial pathogens, may play a causative role in disease through upregulation of inflammatory signals, such as NF-kB, Ras, IL-17, and PI3K, or blunting TNF and IFN γ production in response to these pathogens in the lower airways [12]. In addition, smoking and exposure to indoor and outdoor environmental pollutants are other causes of alterations in the lung microbiota, which can lead to inflammation and diseases such as asthma, COPD, and lung cancer [19,45,46,50,51]. It is unclear, however, if microbial dysbiosis itself is the cause of or a consequence of disease [12].

### 2.1. General Methods to Study the Lung Microbiome

Early studies on the microbiome focused on single, known microbes that could be isolated and cultured. However, in recent years, the application of molecular identification approaches such as sequencing have been widely used to explore entire microbial communities (including microbes that are non-culturable) [19]. Initial molecular techniques used for studying the bacterial microbiome in humans were based on 16S rRNA gene sequencing, which assesses diversity and relative abundance at taxonomic levels [52]. The method is based on PCR amplification and sequencing of the 16S gene encoding bacterial ribosomal RNA, which is a small and highly conserved locus in bacterial DNA, containing nine hypervariable regions (V1–V9) that differ across taxa [47,53,54]. The full-length 16S rRNA gene sequencing provides great taxonomic definition; however, for the detection of the lung microbiome and other body sites, it is more common to amplify one or more of the nine hypervariable regions [55,56,57]. The detection of fungi can be performed similarly, through sequencing targeted regions such as the 18S rRNA gene or internal transcribed spacer (ITS) region [58]. Viruses lack conserved nucleic acid sequences, so in this case, shotgun metagenomics are employed [19,59], which can capture functional information about microbial communities (bacterial, fungal, and viral), allowing for investigating for antimicrobial resistance and virulence genes, and differ from 16S rRNA sequencing, which allows them to analyze diversity and relative abundance and to identify taxonomic groups. However, neither 16S rRNA sequencing nor shotgun metagenomics differentiates between live and dead bacteria [19,47]. Other methods that have been used more recently are metatranscriptomics (RNA sequencing) and metabolomics (small-molecule analysis) depending on living cells, which may better reflect the functional activity of the microbiome [47]. The most common samples used to analyze the lung microbiome are sputum and bronchoalveolar lavage (BAL) fluid. Sputum is a non-invasive method and represents a mix of the upper and lower respiratory tract, but can be problematic due to contamination with oral flora and saliva [60]. On the other hand, BAL, which may contain carryover from bronchoscopy, particularly from supraglottic material, is less influenced by contamination but requires an invasive procedure [47,61].

A bioinformatic analysis is key to the understanding and interpreting of microbial communities. Software such as QIIME2 (Quantitative Insights into Microbial Ecology) [62,63], PICRUSt2 (Phylogenetic Investigation of Communities by Reconstruction of Unobserved States) [64,65], and MaAsLin2 (Microbiome Multivariable Association with Linear Models) [66] has been used to quantify bacteria in the microbiome and predict their functional and clinical impacts, along with others [67,68].

QIIME2 is one of the most commonly applied technologies in a microbiome analysis today [63]. QIIME2 allows users to process raw sequencing data and perform subsequent analyses that provide information about the makeup of the microbial community within a sample. The output from a QIIME2 analysis is a feature table that lists either amplicon sequence variants (ASVs) or operational taxonomic units (OTUs) and the number of observations of each within each sample [63]. This feature table can be used with various QIIME2 plugins to perform taxonomic and microbial diversity analyses, and assess phylogenetic relationships and differential abundance of microbial communities. PICRUSt2 can also use the output of QIIME2 as input. It is a software package that uses an extended ancestral-state reconstruction algorithm to predict which gene families are present and their abundance and then combine gene families to estimate the composite metagenome, using previously published 16S information [64,65]. This allows users to obtain insight into the functional roles of the microbes present in their dataset, especially when metagenomic sequencing is not available, or is not practical to do so [64]. Multivariable associations of microbial features with clinical features can be assessed using the MaAsLin2 package [66].

### 2.2. The Lung Microbiome and the Host Immune System

Compelling evidence from human studies has demonstrated that the respiratory tract is not a sterile environment as previously thought [12,69,70]. The lung microbiome is unique from other microbial communities in the body, such as those in the gut [12], where the gut microbiome can play a role in regulating the host immune system. For the lung, studies have suggested that distinct lung microbial signatures are associated with lower airway immune responses, to prevent uncontrolled and undesirable inflammatory responses, to preserve lung homeostasis [71,72]. These events are mediated by a continuous dialog between commensal bacteria and immune cells resident in the lungs, such as alveolar macrophages (Ams) and dendritic cells (DCs), which can express a range of sensors, called pattern recognition receptors (PRRs), such as Toll-like receptors (TLRs), NOD-like receptors (NLRs), C-type lectin receptors (CLRs), and protease-activated receptors (PARs), which can detect these microorganisms. The same receptors are also involved in the recognition of pathogens and induce a subsequent immune response [72]. These immune cells in the lungs bring into play their immune regulatory properties by inducing the generation of regulatory T cells (*T*_reg_) and by the release of prostaglandin E2 (PGE_2_), tumor growth factor-beta (TGF-β), and interleukin-10 (IL-10) [72]. In summary, evidence indicates that lung microbiota, acting on resident immune cells, have a key role in promoting immune tolerance in the lungs. In a study of patients with severe asthma, *Proteobacteria* species were associated with activation of Th17-associated pathways [73].

In another study, the increased presence of supraglottic-predominant taxa in the lower airway of humans, specifically *Prevotella*, *Rothia*, and *Veillonella*, exhibited a positive association with elevated levels of various Th17 cytokines, including IL-1α, IL-1β, IL-6, fractalkine, and IL-17. This correlation was also observed in conjunction with the enhanced recruitment of both Th17 cells and neutrophils within the lung [70]. On the other hand, it is unclear if the balance between pro-inflammatory effects and regulatory mechanisms becomes altered as aspiration events become more frequent. However, data suggested that persistent exposure to certain microbes can trigger a mechanism that leads not just to increasing inflammation, but also to immune exhaustion [12]. In individuals where the lower airway microbiome is dominated by oral commensals, there is a blunting of the (TLR4) response of alveolar macrophages [73].

In mice, the appearance of bacterial taxa after birth is necessary for the development of *T*_regs_ [74]. Immediately after birth, newborn mice were susceptible to developing excessive airway eosinophilia, accompanied by the release of T-helper two (Th2) cytokines and airway hyper-responsiveness after exposure to house dust mite allergens, although their lungs had great quantities of CD4^+^Foxp3^+^CD25^+^Helios^+^*T*_reg_ cells [74]. During the first 2 weeks after birth, the bacterial load in the lungs increases, paralleled by a progressive shift of bacterial phyla from a prevalence of *Gammaproteobacteria* and *Firmicutes* toward *Bacteroidetes*. The modifications of microbiota composition determine a decreased responsiveness to an aeroallergen due to the appearance of a Helios–*T*_reg_ cell subset that exerts potent immunosuppressive activity cells [74]. The development of this population depends on the increased expression of programmed death-ligand-1 (PD-L1) on DCs, induced by the changes of the lung commensal community. A lack of microbial colonization or PD-L1 blockade during the first 2 weeks after birth caused an excessive sensitivity to allergens that continued until adulthood [74]. Moreover, microbial products, such as short-chain fatty acids (SCFAs), had significant immunomodulatory properties and blunted IFNγ and IL-17 responses to pathogen-associated molecular patterns and exposure of the lower airways to oral commensals, not only triggering an increase in inflammatory cytokines but also an increase in immune-checkpoint inhibitor markers, such as PD-L1, among T cells and recruitment of regulatory T cells [12]. These findings demonstrated how the lung microbiome is important to modulate the innate and adaptive immune system.

Researchers have also found a link between the lung microbiome and brain autoimmunity [75]. Shifting the microbiota toward lipopolysaccharide (LPS)-enriched phyla by local treatment with neomycin induced a type-I-interferon-primed state in brain-resident microglial cells. Their responsiveness toward autoimmune-dominated stimulation by type II interferons was impaired, which led to decreased proinflammatory response, immune cell recruitment, and clinical signs. Suppressing LPS-producing lung phyla with polymyxin B led to disease aggravation, whereas the addition of LPS-enriched phyla or LPS recapitulated the neomycin effect. These findings suggested that dysregulation in the lung microbiome significantly influenced the susceptibility of rats to developing autoimmune diseases of the central nervous system [75]. In addition, it has been found that an intimate relationship exists between the lung microbiome and multiple sclerosis. LPS can cross the blood–brain barrier (BBB) into the brain through blood circulation and influence the development of multiple sclerosis by regulating the microglia in the brain [19]. These findings indicate a close relationship between the lungs and the brain and some authors have already started to refer to it as the lung–brain axis [19].

The association of lung microbiota with the pathogenesis of lung cancer has been reported [19,32,33,51]. A proposed mechanism is that bacteria cause chronic inflammation-promoting factors that stimulate airway epithelial cell proliferation, which induces cell transformation, initiating tumor formation [51]. A study suggested that symbiotic flora of the lungs causes inflammation associated with lung adenocarcinoma by activating γδ T cells that reside in the lungs [51]. These bacteria stimulate myD88-dependent IL-1B and IL-23 production in bone marrow cells, induce proliferation and activation of Vg6^+^Vd1^+^γδ T cells, and mediate inflammation by inducing the production of effector molecules such as IL-17, which may lead to tumor cell proliferation in lung cancer [51]. The incidence of lung adenocarcinoma was also significantly reduced by the elimination of the symbiotic bacteria [51]. The importance of commensal bacteria in supporting the host immune response against cancer has also been demonstrated, revealing a defective induction of lung immunity after antibiotic treatment [76]. Patients with NSCLC have been shown to present significantly higher frequencies of T helper type 1 (Th1) and Th17 cells reacting to *Streptococcus salivarius* and *Streptococcus agalactiae* compared with healthy controls [77]. Moreover, lung inflammation mediated by Th17 cells has been identified as an important factor in the initiation and metastasis of lung cancer [77,78]. However, this finding should be interpreted with caution since it has been shown that Th17-mediated neutrophil responses either promote carcinogenesis or, in contrast, can protect from cancer development and contribute to treatment efficacy [79,80]. The lung microbiome has been shown to have an impact on the host immune system from birth, helping to modulate innate and adaptive systems, and having crosstalk and influencing not just brain autoimmunity, but also the development of other diseases such as lung cancer.

## 3. The Relationship between the Lung Microbiome and Lung Cancer Risk

**Figure 2:**
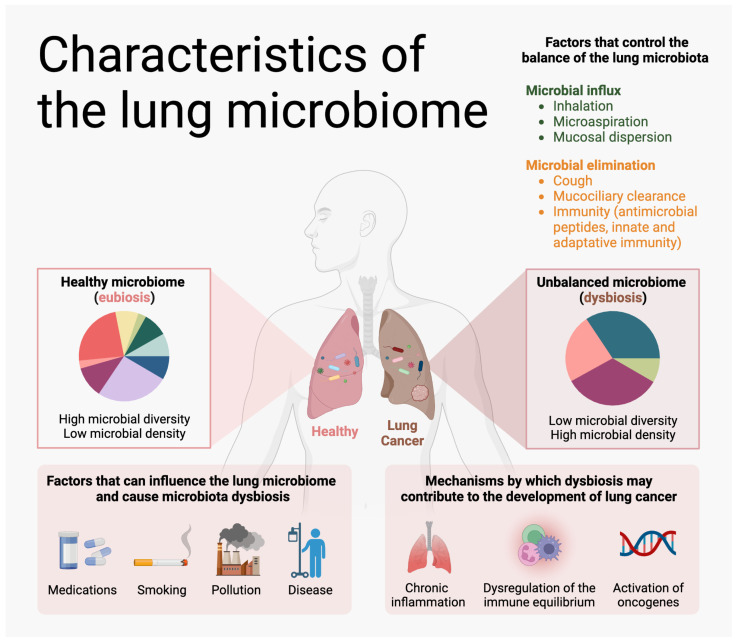
Characteristics of the lung microbiome. The lung microbiome can be altered by several factors, where a healthy microbiome has been observed to comprise a higher number of bacterial species but lower density of those species, and microbial dysbiosis has shown the opposite. Dysbiosis can induce several perturbed health conditions, one being an increased risk of developing lung cancer.

Recently, perturbations in the lung microbiome in the context of lung cancer have been described and may contribute to carcinogenesis. Three mechanisms by which dysbiosis may contribute to the development of lung cancer are (1) the development of chronic inflammation, (2) the dysregulation of the immune equilibrium in the lung, and (3) the activation of oncogenes [81] (Figure 2). In general, the alpha diversity (richness) of the lung microbiome is decreased in lung cancer compared to healthy controls [82,83,84]. Others have reported that although beta diversity (the diversity of the microbiota between different samples) does not drastically differ between lung cancer and controls, dysbiosis of specific microbe species may be a contributor to lung cancer [82,85]. A recent meta-analysis found a significant decrease in the bacterial *Actinobacteria* phylum, *Corynebacteriaceae* and *Halomonadaceae* families, and *Corynebacterium*, *Lachnoanaerobaculum*, and *Halomonas* genera in lung cancer tissue compared to adjacent normal tissue [86]. Phylum TM7 and the genera c:TM7-3, *Capnocytophaga*, *Sediminibacterium*, *Gemmiger*, *Blautia*, and *Oscillospira* were reported to be increased in BAL samples from lung cancer cases compared to controls with benign pulmonary diseases, and this was used to generate a signature to predict lung cancer [87]. Another study found that *Bradyrhizobium japonicum* was present only in BAL from patients with lung cancer but not controls and that *Acidovorax* sp. JS42 and *Acidovorax ebreus* were present in lung cancer and controls with benign pulmonary diseases but not in healthy controls [82]. Additionally, decreased relapse-free survival in lung cancer has been linked to an increase in classes *Bacteroidia* and *Clostridia* and orders *Bacteroidales* and *Clostridiales* in tissue, and increased relapse-free survival has been linked to an increase in classes Alphaproteobacteria and Betaproteobacteria, and orders Burkholderiales and Neisseriales. A recent study using shotgun metagenomic sequencing on BAL samples demonstrated that the rare microbes *Bacteroides pyogenes*, *Lactobacillus rossiae*, and *Burkholderia mallie* were enriched in NSCLC compared to healthy controls [85]. The same study reported age-, sex-, and smoking-specific differences in populations of specific microbes as well as differing microbial populations depending on sampling site, indicating a need to account for these factors when analyzing microbiome differences between cancer and non-cancer.

### 3.1. Smoking

Epidemiologically, it is well known that smoking is a risk factor for the development of lung cancer. Smoking has been shown to alter the lung microbiome in mice [88], and children exposed to second-hand tobacco smoke had decreased alpha diversity and relative increases in *Serratia* spp., *Moraxella* spp., *Haemophilus* spp., and *Staphylococcus aureus* [89]. In adults, smoking is also known to alter the microbiome of the lungs, in particular, by enabling colonization by pathogenic bacteria and thus conferring an increased risk of infections [90]. The immunosuppressive effect of tobacco smoke likely impairs antimicrobial defenses by a variety of mechanisms, creating a permissive environment for colonization by these bacteria [91,92,93,94]. One study has shown that the cigarettes themselves contain bacteria including *Acinetobacter*, *Bacillus*, *Burkholderia*, *Clostridium*, *Klebsiella*, *Pseudomonas aeruginosa*, and *Serratia*, and this could expose smokers to a wide array of potentially pathogenic microbes [95]. Another has shown that exposure to burning coal for household heating leads to increased *Granulicatella*, *Abiotrophia*, and *Streptococcus* in the sputum, expanding the consideration of smoke exposure past tobacco alone [96]. Patients with NSCLC with a smoking history were shown to have increased *Pseudoalteromonas* sp. *CF149*, *Roseburia hominis*, and fungus *Penicillium expansum* and decreased *Pseudomonas mosselii* and *Pseudomonas putida* in BAL samples compared to patients with NSCLC without a smoking history [85]. In contrast, another study found that for lung adenocarcinoma (LUAD) tissue samples, the microbiota abundance and diversity were not significantly different between smokers and non-smokers [97]. These conflicting results may be explained by the difference in lung cancer subtypes, sample type, and/or sequencing method, indicating the need for further standardization of techniques for interrogating and interpreting the lung microbiome.

### 3.2. Chronic Tuberculosis (TB) Infection

Another risk factor for the development of lung cancer that has also been linked to smoking includes chronic tuberculosis (TB) infection. A general overview is that TB causes chronic inflammation of the lung tissue, which can lead to fibrosis and lung cancer [98]. A TB diagnosis comes with an increased risk of lung cancer, which is the highest in the first 5 years after the diagnosis but persists for over 20 years, and was reported in a meta-analysis to be independent of smoking status [99]. There is also a reciprocal relationship between lung cancer and TB in which carcinogenesis and the treatment itself may cause the reactivation of a latent TB infection [100]. The microbiome of the TB lung was investigated using a meta-analysis and was reported to be enriched at the genus level in *Veillonella*, *Rothia*, and *Leuconostoc*, which were unique to TB cases and not present in healthy controls [101]. This study drew from multiple previous studies, which used sputum for TB cases and various respiratory secretions for the healthy controls. Furthermore, the microbiome in TB has been shown to differ from that of lung cancer or healthy controls [102]. Another study compared the microbiome between TB, lung cancer, and pneumonia (caused by *Streptococcus pneumoniae* or *Haemophilus influenzae*) using BAL and found that bacterial alpha diversity was increased in lung cancer compared to TB and pneumonia [103]. The pulmonary microbiome of the TB and lung cancer groups was fairly similar, with only *Mycobacterium* and *Selenomonas* (enriched in TB) and the other two genera, *Sphingobium* and *Marseilla* (enriched in lung cancer), differing. In comparison, TB and lung cancer were both fairly different from the pneumonia controls. One microbiome-related theory behind the mechanism of the lung-cancer–TB relationship is that dysbiosis of the lung microbiome that could be caused by TB infection may lead to the secretion of pro-inflammatory factors that cause chronic inflammation, thus leading to the activation of oncogenes and promotion of tumorigenesis [33,51,103]. Though several studies have also identified *Mycobacterium* or *M. tuberculosis* in the sputum of patients with lung cancer compared to controls [85,104,105], supporting the correlation between TB and lung cancer, it is difficult to tease the relationship apart in the presence of confounding factors such as comorbid COPD or other chronic inflammatory lung disorders.

### 3.3. Chronic Obstructive Pulmonary Disease (COPD)

COPD is a well-known risk factor for the development of lung cancer, and is also a smoking-related disease [106], further highlighting the interplay between lung cancer, smoking, and chronic inflammation. The lung microbiome is also known to be altered in COPD, and an impaired lung microbiome is thought to contribute to the development of COPD [107]. The development of COPD is associated with increased diversity of the microbiome, in particular, of Firmicutes in more severe COPD [107,108]. Another study examining the BAL of patients with lung cancer showed an increased ratio of Firmicutes to Bacteroidetes in patients with lung cancer who were smokers versus patients with lung cancer who were non-smokers, supporting the reciprocal relationship between smoking, COPD, and lung cancer [39]. Another phylum, Proteobacteria, which has been linked to COPD exacerbations and severity [20,109], has also been reported to be enriched in lung cancer [110,111]. Dysbiosis of these two phylums may underpin certain aspects of the mechanistic linkage between COPD and lung cancer.

In summary, the dysbiosis of the lung microbiome has been linked to smoking, TB, and COPD and is seen in lung cancer as well. Smoking is one common linkage between lung cancer, TB, and COPD but so is inflammation and alterations of the immune equilibrium, which may cause dysregulation of the lung microbiome or vice versa. One example is the mechanism by which γδT17 immune cells are modulated by commensal microbes in the lung—disruption of this mechanism by dysbiosis contributes to defective tumor immune surveillance [76]. Another example is the upregulation of specific immune responses (Th1 and Th17) in NSCLC in response to *Streptococcus salivarius* and *Streptococcus agalactiae* compared to healthy controls [77]. The lung microbiome can also influence response to immunotherapy. In NSCLC, the immunotherapy responders (high-PD-L1 group) had increased *Veillonella dispar* compared to the low-PD-L1 group, which was enriched in *Haemophilus influenzae* and *Neisseria perflava* [112]. The influence of the lung microbiome on the immune system, development of cancer, and treatment of cancer by immunotherapy is an incredibly relevant topic that has been recently and thoroughly reviewed [33].

## 4. COVID-19 and Lung Cancer

Coronavirus Disease 2019, or COVID-19, caused by severe acute respiratory syndrome coronavirus 2 (SARS-CoV-2), is a highly contagious infectious disease that has had a devastating global impact, resulting in over 6 million deaths worldwide [113]. The symptoms of COVID-19 range from fever, cough, and headache to sore throat, diarrhea, fatigue, and loss of taste or smell [114]. One of the severe manifestations of COVID-19 is acute respiratory distress syndrome (ARDS), triggering inflammatory events in the lungs of affected individuals [115].

Several studies have explored the microbiome’s role in COVID-19 and its potential impact on cancer, revealing significant findings. Early studies indicate significant changes in the gut microbiome in patients with COVID-19, potentially linking to colorectal cancer pathogenesis [116]. Additionally, it has been demonstrated that patients with COVID-19 develop dysbiotic microbiota, potentially heightening the risk for severe COVID-19 and colorectal cancer [117]. In pancreatic cancer (PC), the alteration of gut microbiota caused by COVID-19 infection showed an impact on PC progression via immune regulation [118]. Recently, it was demonstrated that patients with cancer and COVID-19 have a higher chance of severe symptoms, suggesting an association between the naso-oropharyngeal microbiome, breast cancer, and COVID-19 severity [119]. In patients with lung cancer, the relationship between COVID-19 and lung cancer has also been explored. It has been shown that COVID-19 may alter the tumor microenvironment, promoting cancer cell proliferation and dormant cancer cell reawakening in patients with lung cancer [120]. These cells, reawakened upon infection with SARS-CoV-2, can populate the premetastatic niche in the lungs and other organs, leading to tumor dissemination [120]. These findings emphasize the potential interplay between COVID-19 and lung cancer, necessitating further research for a full comprehension of the implications of COVID-19 on lung cancer and to optimize care for affected individuals.

## 5. Lung Microbiome Profiling as a Method for Early Detection of Lung Cancer

In the past few years, significant efforts have been made to identify indicators of who will develop lung cancer [121,122,123,124], since not all of those deemed ‘high-risk’ such as smokers or those exposed to occupational hazards will develop the disease, and low-dose computed tomography (LDCT) screening of individuals without symptoms is cost-prohibitive. Emerging research suggests that the lung microbiome may play a significant role in lung cancer development [9,16,32,39,104,125,126]. Although there is no agreement on the exact taxonomic classifiers associated with human lung cancer, it is evident that the microbiome plays functional roles in the biological processes involved in cancer biogenesis.

A diverse range of microbial taxa within the lung microbiome have emerged as potential biomarkers for lung cancer. For instance, in patients with lung cancer, the lower airways exhibited an enrichment of oral taxa, including *Streptococcus* and *Veillonella*, correlating with the upregulation of ERK and PI3K signaling pathways [16]. Moreover, metagenomic sequencing of the sputum microbiome identified *Granulicatella adiacens*, along with six other bacterial species (*Enterococcus* sp. 130, *Streptococcus intermedius*, *Escherichia coli*, *Streptococcus viridans*, *Acinetobacter junii*, and *Streptococcus* sp. 6), as a potential non-invasive and innovative biomarker for both lung cancer and its progression [104].

Another study, focusing on the characterization of the microbiome in BAL fluid of patients with lung cancer, highlighted discernible distinctions in bacterial communities between patients with lung cancer and those with benign mass-like lesions. Notably, *Veillonella* and *Megasphaera* exhibited a relatively higher abundance in the former group, suggesting their potential as biomarkers for predicting lung cancer [39]. These studies also underscored the feasibility of analyzing microbial communities from non-invasive samples like sputum and BAL fluid, enabling minimally invasive and repeatable testing.

In a recent groundbreaking study involving 400 patients, encompassing individuals with pre-existing lung cancer, those who later developed the disease, and those who remained cancer-free even after a 10-year follow up, a microbial-based classifier was developed and validated through a linear discriminant analysis. This classifier demonstrated exceptional prowess in predicting lung cancer in patients prior to the clinical diagnosis. This study accentuates the potential of leveraging lung microbiome profiling for the early detection of lung cancer [124].

## 6. Lung Microbiome and Metastasis

**Figure 3:**
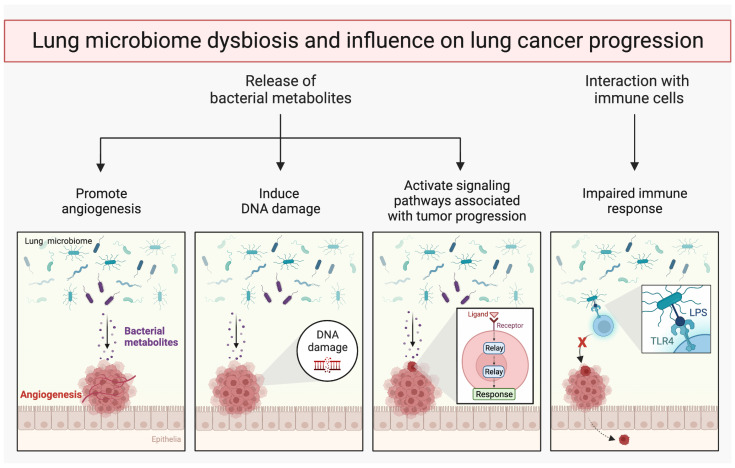
Lung microbiome dysbiosis and influence on lung cancer progression. Microbial dysbiosis in the lungs can alter several vital functions within lung tissue, and can affect cancer cells directly. Some bacteria produce metabolites that can modify vital genes and pathways, causing DNA damage, activating signaling pathways linked to tumor growth, or stimulate angiogenesis. Bacteria can also communicate with immune cells, which can thereby promote cancer metastasis.

The dysbiosis of microbiota has a direct or indirect impact on lung cancer cells, potentially promoting metastasis [127]. One proposed mechanism involves the modulation of the immune response by the lung microbiome. By interacting with the host immune system, the microbiome would influence its ability to recognize and eliminate cancer cells. Disruptions in the composition or dysbiosis of the lung microbiome may lead to an impaired immune response, enabling cancer cells to evade detection and enhancing their metastatic potential [128]. Additionally, certain microbial species or their byproducts can directly influence cancer cell behavior. For example, some bacteria produce metabolites that induce DNA damage, activate signaling pathways associated with tumor progression, or promote angiogenesis, which facilitates tumor growth and metastasis [127] (Figure 3).

Although the field of the lung microbiome and cancer is still in its infancy, prior studies have suggested a connection between the lung microbiome and distant metastasis in lung cancer. For instance, it was demonstrated that Gram-negative bacteria increase NSCLC metastasis via TLR4 activation and mitogen-activated protein kinase phosphorylation [129]. Also, it was found that LRT infection with *Streptococcus* pneumonia enhances the formation of murine H59 NSCLC liver metastases in C57BL/6 mice through host TLR2 activation [130]. Huang et al. found that the α diversity and β diversity of distant metastatic lung cancer and early or local advanced-stage lung cancer were similar [131]. In patients with adenocarcinoma, the authors discovered that the phylum *Firmicutes* and genus *Streptococcus* were significantly increased in lung adenocarcinoma without distant metastasis (AD_M0), compared to lung adenocarcinoma with distant metastasis (AD_M1) [131]. It was also demonstrated that the genus *Streptococcus* could predict distant metastasis of adenocarcinoma. In patients with squamous cell carcinoma, genera *Veillonella* and *Rothia* were significantly increased in lung squamous cell carcinoma with distant metastasis (SCC_M1), compared to lung squamous cell carcinoma without distant metastasis (SCC_M0). Thus, genera *Veillonella* and *Rothia* could serve as biomarkers in predicting distant metastasis of squamous cell carcinoma [131]. Yu et al., by profiling the lung microbiota of 165 non-malignant lung tissue samples from patients with cancer, found that the genus *Thermus* (*Thermi*) is more abundant in tissue from patients at an advanced stage (IIIB, IV), while *Legionella* is higher in patients who develop metastases [111].

While these studies provide intriguing evidence, it is important to note that the exact mechanisms linking the lung microbiome and the metastatic potential of lung cancer cells are not yet fully understood. Further research is needed to elucidate the specific microbial species, interactions, and pathways involved. Furthermore, understanding how microbiome-influenced metastasis affects specific organs, such as the brain and bones, may provide important insights into how lung microbiome dysbiosis affects cancer progression in specific organs.

## 7. Intratumoral Microbiome

The tumor microenvironment is a complex ecosystem where tumor cells coexist with various immune cells like macrophages, polymorphonuclear cells, mast cells, natural killer cells, dendritic cells (DCs), T and B lymphocytes, and non-immune cells such as endothelial cells and stromal cells. These cells establish subtle interactions with each other that can either promote or inhibit tumor growth and invasion [132,133]. Recently, the tumor microbiome, another important component of the TME, has attracted significant attention [134,135,136,137]. The intratumor microbiome is a major constituent of the TME, wielding a substantial influence on various aspects of cancer dynamics, including the tumorigenesis, disease progression, drug resistance, and prognosis [138,139,140]. Nejman et al. surveyed 1010 tumors for bacteria across melanoma, lung, ovarian, glioblastoma, pancreas, bone, and breast cancers [141]. The study revealed significant differences in composition, diversity, and metabolic functions encoded by intratumor bacteria between cancer types. Histologic imaging revealed heterogeneous microbial spatial distributions and their frequent intracellular localization in cancer and immune cells [141]. The tumor microbiome refers to the community of bacteria, viruses, fungi, and other microorganisms within the TME [134]. Although it is a relatively new field of study, it holds significant potential for enhancing the understanding of cancer development, progression, and treatment [137,142]. Studies have shown the pivotal role of the intratumor microbiome in influencing local inflammation [143], immune responses [136], and cellular metabolism [144] within the TME.

### 7.1. Metabolic Interactions between Tumor Cells and Microbiome

Both cancer and immune cells heavily rely on specific nutrients and metabolites, such as glutamine, glucose, arginine, and asparagine [145,146]. Microbial cells within the TME exhibit a remarkable capability to produce a diverse range of metabolites. These metabolites encompass bioactive molecules such as short-chain fatty acids (SCFAs), amino acids, and vitamins [147,148]. These compounds, often derived from microbial fermentation of dietary substrates, have the potential to modulate cellular processes in the local environment [149]. It has been demonstrated that high concentrations of fecal or plasma SCFAs like acetate, propionate, or butyrate (major metabolites of microbial starch degradation) were observed to stratify progression-free survival in patients with cancer treated with anti-PD-1-type immune checkpoint inhibitors [150]. Short-chain fatty acids exhibit immunomodulatory functions in the host, affecting CD4^+^ T cells and antigen-presenting cells [150]. Of particular interest are the potential consequences of these microbial metabolites on adjacent tumor cells. In lung cancer, it is becoming increasingly evident that these metabolites may act as essential nutrients, providing energy sources for tumor cell growth and proliferation [144]. Furthermore, bacteria enriched in lung carcinomas may potentially possess an ability to metabolize cigarette-associated metabolites [141]. Further, it has been shown that bacteria are present within tumor cells, suggesting that bacteria could indeed be influencing cancer cell signaling from inside the cell by local nutrient provisioning [141]. In fact, accumulating evidence supports the notion that a metabolic dependence exists between the tumor cells and the cells in the stroma [151,152]. Although it has been discussed how the metabolism of nearby, non-cancerous cells affects cancer cell metabolism and growth in the TME [153], the relationship between microbial cells and tumor cells is still unclear. On one hand, microbial cells could thrive on nutrients derived from the TME, generating metabolites that could enhance the survival of neighboring tumor cells. Likewise, tumor cells could release waste products that microbial cells utilize as substrates. This contributes to the intricate interdependence between these entities [135,153,154,155]. The complexity increases when we consider the metabolic communication between tumor cells and immune or stromal cells in the TME, and the organ-specific metabolite composition [153].

These metabolic interactions within the TME extend their influence on immune cells, influencing their metabolic profiles, activation, and effector functions [156,157,158]. Microbial metabolites are emerging as potential regulators of immune responses against tumors [136,150]. Moreover, manipulating microbial communities in the TME could reshape the metabolic landscape, providing new therapy opportunities for lung cancer.

### 7.2. Metabolites Produced by Microorganisms Can Promote Cancer Development

Metabolites are biological molecules generated as a result of cellular metabolism. These molecules can function as signaling molecules or modulate cellular activities in response to changes in metabolic processes [159]. Increasing evidence shows that metabolites produced by a range of human-associated microorganisms within several types of cancer can influence its progression and resistance depending on the cancer niche [154,160,161].

Some examples of these metabolites include methylglyoxal (MGO) and SCFAs like butyrate and lactate, which can influence chromatin architecture, either promoting or suppressing cancer. Secondary bile acids (sBAs) from gut microbiota metabolism, especially deoxycholic acid (DCA) and lithocholic acid (LCA), can stimulate colorectal cancer progression. Microbial polyamines are associated with prostate cancer inhibition and more specifically polyamine cadaverine can inhibit the epithelial–mesenchymal transition [161,162,163].

Although the link between metabolites produced by microbes and lung cancer development is poorly understood, there are associations between microbiome-induced inflammatory processes and lung cancer. For instance, microcystin produced by cyanobacteria, commonly enriched in lung adenocarcinoma, can lead to increased expression of procyclic acidic repetitive protein 1 (PARP1), inducing inflammation [164]. Furthermore, emerging studies point to a gut–lung axis, where neoplastic transformation and lung cancer progression may also be linked to gut dysbiosis [165]. Recently, Vega et al. demonstrated that the local tumor microbiome is potentially a source of methionine that can directly impact tumor progression [144]. Further investigations are required to elucidate these complex relationships and their implications for lung cancer prevention and treatment.

## 8. Microbiome in Lung Cancer Treatment

Lung cancer is a molecularly heterogeneous disease, which influences not only tumor progression but also the composition of the TME and, therefore, its microbiome [2]. As with other cancers, patients with lung cancer likely have a unique microbial signature, and more than 15 specific tumor-associated microorganisms have been associated with lung cancer. However, the precise influence of these microorganisms on disease progression and resistance is still an area requiring further investigation [166,167].

Recent studies suggest the microbiome may be a promising target for lung cancer treatment [32,41,168,169]. Manipulating the microbiome may be a potential strategy to enhance lung cancer treatment effectiveness, such as improving the efficacy and patient response to immunotherapies, while simultaneously mitigating therapy-related side effects like dysbiosis. Furthermore, it can be implemented as biomarkers for personalized medicine and disease prevention [166,168,169,170].

One recent study investigated the gut microbiota and established a distinct gut microbial profile for the potential prognosis of early-stage lung cancer [166]. By knowing the patient’s microbial profile, an approach is to target microbiome-derived metabolites that can impact lung cancer development or host immune cells’ anti-cancer activity by TME modulation. In a non-cancer example, NOD mice were fed acetylated or butyrylated starch, which appeared to lower levels of autoimmune T cells and increase the number of *T*_reg_ cells, overall reducing the incidence of diabetes in these mice [171]. This highlights the potential for these bacterial metabolites to influence the immune response and be applied in a therapeutic manner. Indeed, microbial SCFAs have been shown to play an important role in anti-tumor immunity and perhaps could also be exploited for use in cancer therapies [172]. These techniques can potentially undergo testing in experimental tumor models, aiming to achieve therapeutic outcomes and optimal distribution of SCFAs [156,172].

In cancer immunotherapy, particularly NSCLC, Programmed Death 1 (PD-1) inhibitors are frequently used [32]. Derosa et al. demonstrate that the relative abundance of the gut bacterium *Akkermansia muciniphila* can predict the clinical response of patients with NSCLC to the PD-1 blockade. Furthermore, mice that received fecal microbial transplants (FMTs) negative for *A. muciniphila* demonstrated tumor resistance to the PD-1 blockade [173]. This phenotype was rescued with oral supplementation of the immunogenic strain of *A. muciniphila*, Akkp226118 [173]. Similarly, oral supplementation of *A. muciniphila* after FMT with nonresponder feces restored the efficacy of the PD-1 blockade by recruiting CCR9^+^CXCR3^+^CD4^+^ T lymphocytes into tumor sites [174].

In NSCLC chemotherapy, gemcitabine is a drug commonly used. The gammaproteobacteria *E. coli* has been shown to reduce gemcitabine efficacy in in vitro assays and possess a cytidine deaminase (CDA), such as *Mycoplasma* bacteria, which can break down gemcitabine. Furthermore, there is a strong association between mycoplasma infection and carcinogenesis, and almost all surgically removed lung cancer samples present mycoplasma infection [32]. Understanding and mitigating these microbial influences help tailor more efficient treatment strategies. However, although these are compelling pieces of evidence paving the way in lung cancer treatment using the microbiome and its products as an allied tool, many questions remain unanswered about this complex relationship and deserve further investigation.

## 9. Limitations and Future Directions

The field of research on the lung cancer microbiome has made significant advances in recent years; however, it still faces various challenges that require attention in future studies. While the role of the gut microbiota in the development of digestive system cancers [175,176,177,178,179,180], and cancer treatment response, has been widely studied [160,181,182], the identification of microbes within solid tumors is a relatively new concept, with limited studies in this area. Putting more effort into these studies can help with better understanding their role in cancer occurrence and progression, with potential therapeutic and diagnostic applications, making it a promising novel strategy to inhibit tumor development and enhance therapeutic efficacy. New observations implicate their involvement in tumor development and the role of tumor metabolism. Given that the TME, including the microbiome, is a complex pathological ecosystem [183], cooperative and competitive relationships among microbes and tumor cells may influence tumorigenesis and cancer progression [184,185]. For example, bacteria may influence cancer cell signaling from within by locally providing nutrients [141]. Simultaneously, while supplying metabolites to cancer cells, they compete for nutrients in the nutrient-poor tumor microenvironment [135]. Understanding these interactions could offer insights into novel therapeutic strategies for cancer treatment and microbiome-based interventions.

There are concerns about the accuracy, prevalence, and consistency of the intratumor microbiome during cancer treatment that need addressing before clinical applications [134]. Moreover, studying the lung microbiome and intratumor microbiome and their effects on tumorigenesis, disease progression, and treatment outcomes is challenging due to the low-biomass microbial populations in the lungs [134]. Additional studies will help reduce false positives and clarify the biological relevance of these tumor microbiome interactions. Furthermore, the analysis of the microbiome is affected by various experimental conditions and computational challenges [186]. While next-generation sequencing techniques have greatly improved the understanding of the roles of microorganisms, they are susceptible to issues such as sequencing errors, genomic repeats, and computationally intensive downstream analyses. The introduction of new sequencing technologies and protocols has added complexity, influencing the outcomes of analyses [186]. To address the challenges associated with a microbiome data analysis, researchers have developed various workflows and protocols for sequencing 16S rRNA, shotgun, and long-read metagenomics. These frameworks aim to streamline and standardize the computational analysis of microbiome data by addressing key aspects such as experimental design, sample processing, sequencing, assembly, binning, annotation, and visualization [186].

Finally, clinical trials, such as “Microbiota and the Lung Cancer (MICA)”, (NCT03068663), have been initiated to investigate the role of the microbiota in lung cancer. These trials aim to explore the clinical applications of microorganisms in lung cancer treatment, decipher the interplay between lung cancer and the lung/gut microbiota, and evaluate the microbiota as a potential therapeutic target for lung cancer [187]. These clinical trials, focused on the relationship between the lung microbiota and lung cancer, play a crucial role in advancing the understanding of the microbiome’s involvement in lung cancer initiation, progression, and treatment outcomes. They hold the potential to offer valuable insights into the clinical applications of microorganisms in lung cancer treatment and the development of microbiota-based interventions for improved therapeutic outcomes.

## 10. Conclusions

In conclusion, it is clear that the microbiome has a crucial role in biological processes related to the development and progression of lung cancer. Metagenomic sequencing can be utilized to identify the microbial population that resides in the airway, providing a promising method to detect changes in the lung microbiome as potential indicators of cancer development, especially among high-risk groups like current and former smokers. Although research on the potential of the lung microbiome as a source of biomarkers for lung cancer is still in its early phases, research findings indicate that the microbiome holds promise for this purpose. Further investigations are required to gain a deeper understanding of the role of the lung microbiome in the development of lung cancer and to identify specific microbial biomarkers as clinically applicable tools to improve early diagnoses and treatment.
